# Physical and Mechanical Properties of Bulk-Fill, Conventional, and Flowable Resin Composites Stored Dry and Wet

**DOI:** 10.1155/2022/7946239

**Published:** 2022-02-10

**Authors:** Dana Jafarpour, Reihaneh Ferooz, Maryam Ferooz, Rafat Bagheri

**Affiliations:** ^1^Private Practice, Shiraz, Iran; ^2^La Trobe University, Melbourne, Australia; ^3^General Dentist, Williamtown, Australia; ^4^Department of Dental Materials, Biomaterials Research Centre, Shiraz Dental School, Shiraz University of Medical Sciences, Shiraz, Iran

## Abstract

Surface degradation, margin, and bulk fracture are common reasons that necessitate replacement of resin composite restorations. The purpose of this study was to determine filler weight (FW), fracture toughness (FT), Vickers hardness (VHN), sorption/solubility (S/S), and colour change (ΔE) of four resin composites in dry and wet conditions. Four resin composites of shade A2 were investigated: Aura bulk-fill (AB) (SDI), Tetric Evoceram (TE) (Ivoclar), G-ænial Universal Flo (GUF) (GC), and GC Kalore (GCK) (GC). For FT, VHN, and ΔE, the specimens were prepared, divided into 2 groups, and stored dry or immersed in distilled water. The specimens were subdivided into three subgroups and stored for 1, 7, and 60 days and then subjected to the relevant tests. Six fractured remnants were weighed for each material to measure FW%. To test S/S, ISO 4049 was used. The data were analysed using ANOVA and Tukey's test. There was an inverse correlation between FW and FT. A significantly higher FT was found for GUF. There were no significant differences between conditions in materials except for AB. The highest VHN was found for GCK and AB. After 1 and 7 days, a significant difference was observed in S/S between all materials with the highest values for GUF. There was a correlation between sorption and solubility. The material, the media, and aging have an influence on the properties of resin composites. It is important to emphasise that each material should be used for a specific clinical need based on their properties.

## 1. Introduction

Among direct tooth-coloured dental restorative materials, resin based composite shows an appropriate combination of aesthetic and mechanical performance. Since their widespread application as posterior restorations in the 1990's, manufacturers and researchers have focused on improving mechanical and physical properties of resin composites [1]. Resin composites must show resistance to environmental factors such as masticatory forces, occlusal habits, abrasive food, chemically active food and liquids, temperature fluctuations, humidity variations, bacterial products, and salivary enzymes [2].

Fracture toughness of a brittle material describes its resistance to crack propagation and is an inherent feature of a material indicated by K_Ic_ [3]. To measure fracture toughness, different test methods have been established including four-point bending. Fujishima and Ferracane [4] concluded that the four-point test was the most indicative of “true” fracture toughness among all tested methods, as it provided the most information about crack initiation and propagation in a dental composite. Nevertheless, Vickers microhardness, defined as a materials resistance to indentation, is among the physical properties that governs a resin composite's clinical performance [5]. Surface degradation, margin, and bulk fracture are common reasons that necessitate replacement of restorations [6]. Among the factors that affect surface hardness and fracture toughness of resin composite are the quality and technique of polishing, chemical composition such as organic matrix chemical components, filler loading [7], and aging in water and various other media [8].

Filler loading of resin composites greatly determines its mechanical and physical properties [9–11]. However, specifications provided by manufacturers regarding filler load have been shown to be inaccurate in certain instances [7]. Evaluating the filler content by ash method might provide more reliable measurement for research purposes. Hence, the authors decided to measure the filler weight of the tested composites using the standard ash method. The results will help to improve the physical and mechanical performance of resin composites by detecting potential causes of failure for the long-term structural integrity and reliability of resin composite restorations.

A positive correlation between filler loading and surface hardness was established by Kim et al. [7] in an extensive study on filler morphology, its influence on filler loading, and the effect of these factors on the mechanical properties of resin composites. The composites were classified into 4 categories according to filler morphology, which was found to affect filler loading. Composites containing round particles had the highest filler content and the highest hardness. This study utilized standard ash method to measure filler weight content wherein specimens were weighed, heated in order to burn out the organic matrix, and reweighed. The second weight measurement denotes inorganic filler weight [7].

Another study [12] confirmed the positive correlation between filler loading and microhardness in their study on the effect of aging in three food simulating solvents on eight commercial resin composites including four bulk-fill, two microhybrid, and two nanohybrid resin composites. Vickers microhardness reduced significantly, mainly as a result of storage time and type of solvent used. Further measurements were taken at baseline and after 7, 30, and 90 days. Additionally, nanohybrid materials showed better mechanical properties and higher hardness values compared to other tested materials, which was speculated by the higher filler loading [2].

However, other researchers [13] investigated the effect of accelerated aging on the Knoop microhardness of 5 light curing and 5 chemically curing resin composites and found a significant increase in hardness following the aging process. They found no correlation between the hardness and the filler content, or between hardness and degree of colour change of the resin composites [13].

Another study [14] on the effect of distilled water on the microhardness of two microhybrid resin composites, Charisma (Dentsply) and Filtek Z250 (3M ESPE), determined a significantly lower hardness of the materials after 6 months of immersion. However, with similar filler loading yet different matrix content compared to Charisma, Filtek Z250 showed higher surface hardness [14], confirming that resin matrix content could be a responsible factor for water sorption [12]. Thus, considering previous studies, it can be confirmed that water uptake increases following an increase in the TEGDMA content in the resin matrix. Charisma with higher amounts of TEGDMA presents higher water sorption when compared to Bis-GMA and UDMA containing resin composites [15].

Water sorption and solubility are significant properties of dental materials, which can be used for predicting the clinical success of resin composites [16, 17]. While a small water uptake may compensate marginal gaps produced by polymerization shrinkage through increasing the bulk volume of restorations [18, 19], a greater expansion than the polymerization shrinkage is undesirable due to the potential expansion stress inducing microcracks or even macrocracks in restored teeth [20]. Additionally, water sorption leads to leaching of some components from resin composites, resulting in further shrinkage, reduced bulk, weakened mechanical properties [21], and compromised biocompatibility [16]. Moreover, water sorption and solubility are considered determining factors of dental materials' colour susceptibility [22]. In a study by Alshali et al. [16] on the hygroscopic changes of six bulk-fill and eight conventional resin composites immersed in distilled water and artificial saliva up to 360 days, a correlation between filler loading and water sorption was established. It was proposed that the sorption of resin composite was negatively correlated to its filler wt. % loading. Due to having a higher depth of cure and relatively high mechanical properties, bulk-fill composites are recommended to be used for postendodontic reconstruction [23].

Although previous studies have compared mechanical and physical properties of different types of resin composites, none of them have measured the filler content using the ash method comparing physical and mechanical properties of bulk-fill, conventional, and flowable resin composites stored wet and dry. Therefore, this in vitro study aimed to measure filler weight (FW), fracture toughness (FT), Vickers hardness (VHN), sorption/solubility (S/S), and colour change (ΔE) of two bulk-fill, one conventional, and one flowable resin composites in dry and wet conditions up to 60 days.

The null hypothesis was that there was no difference among the materials, filler weight has no effect on physical and mechanical properties of the resin composites, storage condition does not affect those properties, and aging does not influence the physical and mechanical properties of dental resin composites.

## 2. Materials and Methods

Four resin composites of shade A2 were tested as presented in Table 1.

### 2.1. Filler Weight Measurement

The filler weight percentage of resin composites was determined by the standard ash method [7]. For each resin composite, six fractured remnants of the fracture toughness control (dry) group were weighed. The specimens (*W*_0_) were weighed on an analytical calibrated electronic balance (GR-300, A&D Company, Toshima-ku, Tokyo, Japan) with an accuracy of ±0.1 mg. The specimens were heated in an electric furnace (heating furnace; Kousha Fan Pars Co., Tehran, Iran) at 600°C for 30 minutes to burn out the organic matrix and were then reweighed (*W*_1_). Filler weight fraction (wt. %) was determined using the following formula:

(1) $$\begin{array}{c} \text{filler wt.}\%=\left(\frac{W_{1}}{W_{0}}\times 100\%\right). \end{array}$$

### 2.2. Fracture Toughness Test

A custom-made, brass and aluminum mould with a centrally placed notch was used to prepare 60 rectangular notched beam specimens with the dimensions of 30 mm × 5 mm × 2 mm for each material. The mould was filled with the material and covered with a plastic matrix strip, gently pressed between two glass slabs to extrude excess materials. The specimens were cured using LED curing machine (Radii plus LED; SDI, Bayswater, Vic, Australia), with a wavelength of 440–480 nm and an output of 1500 mW/cm, according to the manufacturers' instructions. In order to obtain a flat surface, the edges of the specimens were moistened and gently ground manually with P1000 and P1500 silicon carbide papers and rinsed in between. A new razor blade was used with hand pressure to create a sharp crack in the notch. Crack length (d) was measured using a Stereo microscope (BestScope BS-3060C; Beijing, China) at ×80 magnifications. The width and the height of each specimen were measured using a digital caliper (Absolute Caliper; Mitutoyo Kawasaki, Japan). Specimens were randomly divided into 2 groups and stored at 37°C dry or immersed in distilled water. In each group, the specimens were subdivided into three subgroups (*n* = 10) and stored for 1, 7, and 60 days.

After each time interval, the specimens were tested for fracture toughness by placing them in a 4-point test jig and loaded at a cross-head speed of 0.5 mm/min using a universal testing machine (Zwick/Roll Z020; Zwick GmbH & Co, Germany). The maximum failure load was recorded and the K_Ic_ (MPam^0.5^) calculated using the following formula:

(2) $$\begin{array}{c} K_{1c}=\frac{F_{c}}{R\sqrt{W}}\cdot \frac{S_{1}-S_{2}}{W}\cdot \frac{3\sqrt{\alpha }}{2{\left(1-\alpha \right)}^{1.5}}\cdot Y, \end{array}$$

where *F*_*c*_ is fracture load, *S*_1_ is outer span, *S*_2_ is inner span, *R* is specimen width, *α* = *a*/w, *α* is notch depth, *w* is specimen height, and *Y* is stress intensity shape factor:

(3) $$\begin{array}{c} Y=1.9887-1.326\alpha -\left(3.49-0.68\alpha +1.35\alpha^{2}\right)\alpha \left(1-\alpha \right){\left(1+\alpha \right)}^{-2}. \end{array}$$

### 2.3. Vickers Microhardness Test

For each material, 60 disc-shaped specimens were prepared using a polyethylene mould of 10 mm in diameter and 2 mm in thickness and cured according to the manufacturer's instruction using the same light-curing machine explained above. The specimens were divided into two groups of 30 and stored in wet or dry conditions for one, seven, and 60 days (*n* = 10) and then tested for Vickers microhardness. Each specimen was subjected to 3 indentations (*n* = 10 × 3 = 30) 35 *μ*m apart across the surface by applying a load of 0.3 kg for 15 seconds using a digital hardness tester (MHV-10002, SCTMC, China) and the average was recorded as the Vickers microhardness number (VHN).

### 2.4. Sorption and Solubility Test

Disc shaped specimens (*n* = 10) were prepared for each material using a polyethylene mould by the procedure detailed in ISO 4049 [24] as described below. The mould, measuring 10 mm in diameter and 2 mm in thickness, was filled with the material and enclosed by two Mylar strips to avoid exposure to oxygen during curing. These were then pressed between 2 glass slabs using gentle hand pressure to extrude excess material and minimize porosities. The specimen was cured using the same LED light-curing unit on both sides. The specimen was gently removed from the mould and visually inspected for visible flaws, and any excess flash was removed using an abrasive paper. Following polymerization, the specimens were weighed using an analytical calibrated electronic balance (GR-300, A&D Company, Toshima-ku, Tokyo, Japan) to an accuracy of 0.1 mg and transferred to a desiccator with fresh silica gel and stored at 37°C for 24 h. The specimens were weighed after 24 h and repeatedly every 8 h until constant mass was attained (i.e., the mass loss of each specimen was not more than 0.1 mg). This measurement was denoted as *m*_*0*_. The volume of the specimen was also recorded using a digital caliper (CD-8”CSX, Mitutoyo Corp., Japan) to an accuracy of 0.1 mm. The mean diameter was calculated by measuring the diameter of each sample at two points at right angles. The mean thickness was obtained by measuring the thickness at 5 equally spaced points on the circumference of the sample. The volume was then calculated using the following equation:

(4) $$\begin{array}{c} V=\pi r^{2}h, \end{array}$$

where *V* is volume, *r* is the radius, and *h* is the thickness of the specimens.

After baseline measurements of weight and volume were obtained, the specimens were placed into custom racks made of clear radiographic films and immersed in distilled water. The samples were vertically positioned and had more than 3 mm of space between them. The volume of water was considerably more than 10 ml per specimen, as is advised by ISO 4049. Weight was measured at 1, 7, and 60 d of water storage. At each measurement, specimens were dried until free from visible moisture and then weighed with the recorded mass denoted as *M*_1_.

After 60 days, the specimens were transferred to a desiccator containing silica gel. Weight was measured at 1, 7, and 60 days of desiccation and denoted as *M*_2_. Sorption and solubility were calculated using the following formulas:

(5) $$\begin{array}{c} \text{SO}=\frac{\left(M_{1}-M_{2}\right)}{V},\\ \text{SL}=\frac{\left(M_{1}-M_{2}\right)}{V}, \end{array}$$

where SO is sorption, SL is solubility, *M*_0_ represents the specimen's mass before immersion, *M*_1_ is the mass after immersion, *M*_2_ is the mass after desiccation, and *V* is the specimen's volume before immersion.

### 2.5. Colour Stability

The specimens were prepared following the same protocol used for microhardness (*n* = 20 for each material. In this study, Standard Commission Internationale de L'Eclairage (CIE Lab) was used for colour measurement. The CIE *L*^*∗*^*a*^*∗*^*b*^*∗*^ colour system is a three-dimensional colour measurement. *L*^*∗*^ represents the value of an object, ranging from white at the top (100) and black at the bottom (0); *a*^*∗*^ and *b*^*∗*^ are chromaticity coordinates along the red-green and yellow-blue axes, respectively. Coordinate *a*^*∗*^ measures red (C) at one end, green (K) at the other, and grey in the middle (0). Similarly, coordinate *b*^*∗*^ measures yellow (C) at one end, blue (K) at the other, and grey in the middle. The ∆*E* value represents relative colour changes that an observer might report when evaluating aesthetic restorative material. A colour measurement was conducted for each specimen using a spectrophotometer (Spectroshade; MHT Optic Research AG, Zurich, Switzerland) over a white background. The spectrophotometer was calibrated over white and black backgrounds. Once photocured, the specimens were randomly divided into 2 groups and stored at 37°C dry (*n* = 10) or immersed in distilled water (*n* = 10). In each group, the specimens were stored for 1, 7, and 60 days. The water medium was changed weekly. After each time interval, colour measurements and CIE (*L*_0_, *a*_0_, *b*_0_) parameters were recorded. The colour change (∆*E*_*n*_) was calculated using the following formula:

(6) $$\begin{array}{c} \Delta E_{n}={\left[{\left(\Delta L_{n}\right)}^{2}+{\left(\Delta a_{n}\right)}^{2}+\left(\Delta b_{n}^{2}\right)\right]}^{1/2}. \end{array}$$

The differences were determined as ∆*E*_1_ (1–7 days), ∆*E*_2_ (7–60 days), and ∆*E*_3_ (1–60 days).

### 2.6. Statistical Analysis

The collected data was analysed using the statistical software (version 21, SPSS Inc., Chicago, IL, USA) and *P* ≤ 0.05 was considered as significant for all tests. Kolmogorov–Smirnov test was employed to assess normality assumption. The normality assumption was held in all cases. Three-way and two-way ANOVA were used to evaluate interactions between variables (materials, storage times, and conditions). One-way ANOVA with post hoc Tukey's test were used for subgroup analysis of fracture toughness, VHN, and colour stability in each group. For measurement of sorption and solubility, the mass change data was analysed by repeated measures ANOVA. For comparison of different variable between wet and dry conditions, independent *t-*test was used. The Pearson correlation coefficient was performed to evaluate the possible correlation between the fracture toughness, sorption/solubility, VHN, colour change, and filler weight.

## 3. Results

### 3.1. Filler Weight

Filler weight was measured and an average was calculated for each material. The data are represented in Table 1. According to the findings, GUF had significantly lower filler weight (62.82%) compared to other resin composites.

### 3.2. Fracture Toughness

**Figure 1:**
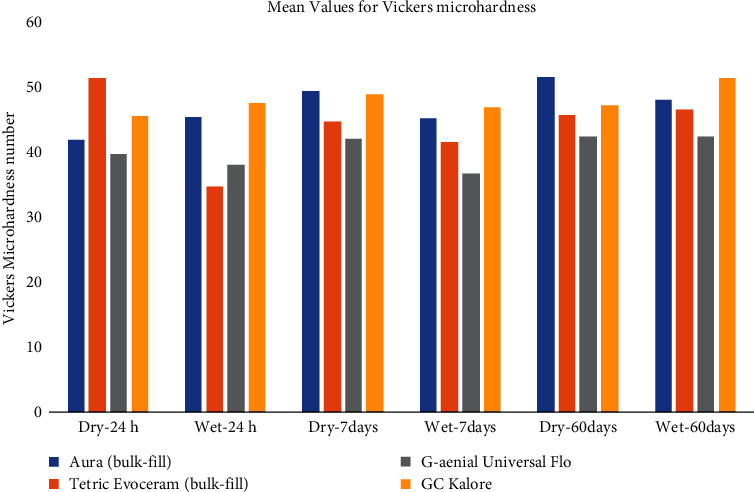
Mean values for fracture toughness of all materials.

The interaction between time, material, and condition was assessed and only the relationship between time and material was statistically significant (*P* < 0.05). For various storage times and in both conditions, fracture toughness of GUF was significantly higher than other materials (*P* < 0.05) except for 24 h storage in wet condition wherein no statically significant difference was found between materials (*P*=0.257) (Table 2 and Figure 1).

Comparison of fracture toughness between wet and dry conditions revealed no significant difference between conditions in TE, GUF, and GCK (*P* > 0.05). However, for AB, fracture toughness was significantly lower in wet condition compared to dry after 7 and 60 days (*P*=0.009 and *P*=0.001, respectively) (Table 2).

### 3.3. Vickers Microhardness

**Figure 2:**
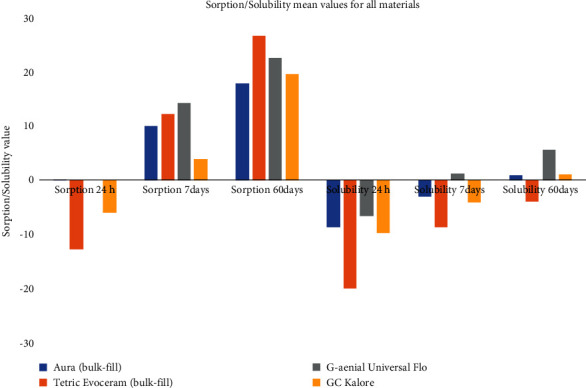
Mean values for Vickers microhardness of all materials.

The interaction between time, material, and condition was assessed and all interactions were statistically significant (*P* < 0.05). As shown in Table 3 and graphically in Figure 2, there was a significant difference between materials in both conditions and in all time intervals (*P* < 0.001). In wet condition, GCK had the highest VHN followed by AB, TE, and GUF in all storage times. In dry condition, the VHN of TE (51.29 ± 2.76) was significantly higher than other resin composites only for 24 h (*P* < 0.001). For the remaining time intervals in the dry condition, AB had the highest VHN followed by GCK, TE, and GUF.

To compare different conditions, dry storage led to significantly higher VHN compared to the wet condition in TE after 24 h (*P* < 0.001), in all materials after 7 d (*P* < 0.05), and in AB after 60 days (*P* < 0.001). However, dry stored specimens of AB in the first 24 h and GCK following 24 h and 60 days of storage showed significantly lower VHN compared to the wet conditions (*P*=0.018, *P*=0.036, and *P*=0.005, respectively).

### 3.4. Sorption and Solubility

**Figure 3:**
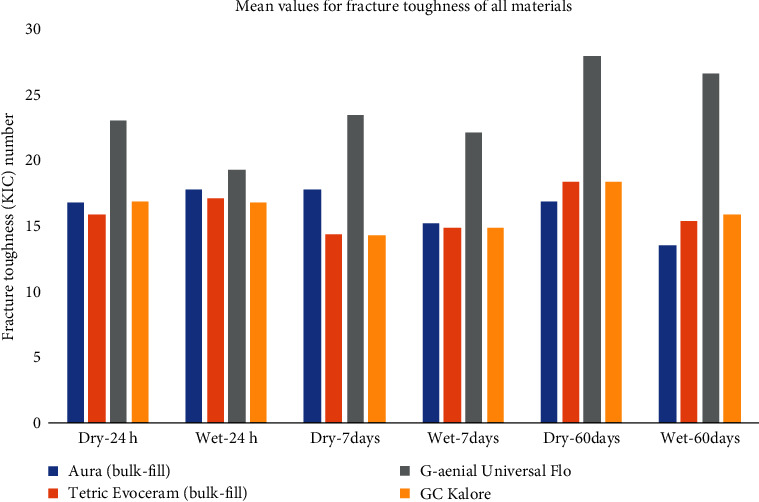
Mean values for sorption/solubility of all materials.

There was a significant difference in the sorption values of the various materials after 1 (*P* < 0.001) and 7 days (*P* < 0.001), with the highest values for GUF (Table 4 and Figure 3). Moreover, 60 days of storage in water led to a significant increase of water sorption values in all materials (*P* < 0.05). Significant differences were observed between the water solubility values of all materials in all storage times (*P* < 0.001) with GUF having the highest amount of solubility.

### 3.5. Colour Stability

For the colour change measurement, two-way ANOVA revealed a significant interaction among the materials and conditions (Table 5). In almost all materials, wet stored specimens revealed a higher value for ∆*E* than the dry specimens, with the finding being significant in bulk-fill composites (*P* < 0.05). However, in GUF, dry specimens showed higher colour change compared to wet conditions after 7 and 60 d (*P*=0.218 and *P*=0.002, respectively). In dry conditions, there was no significant difference between materials in ∆*E*_1_ (*P*=0.248), yet statically significant differences were found between materials in ∆*E*_2_ and ∆*E*_3_ in both dry and wet conditions (*P* < 0.05). After 60 d in both conditions, AB showed the highest and GCK had the lowest ∆*E*_3_ values among tested composites.

### 3.6. Correlations

There was a strong correlation between sorption and solubility (*r*^2^ = 0.840, *P*=0.001). Fracture toughness showed a strong inverse correlation with filler weight (*r*^2^ = −0.777, *P*=0.003). Conversely, filler weight showed no correlation with sorption, solubility, colour change, or microhardness. In addition, microhardness had no correlation with sorption, solubility, colour change, and fracture toughness (Table 6).

## 4. Discussion

The results of this in vitro study rejected some aspects of the null hypotheses; supporting that the type of the material, condition and time intervals have an influence on the physical and mechanical properties of dental resin composite material while filler wt. % had a significant effect only on fracture toughness. The properties of resin composites mainly rely on their monomer type, filler size, filler volume, and filler-resin interface [25].

Different new and popular materials were selected in this study to represent different categories of resin composites (bulk-fill, flowable, and conventional) and to determine the differences in their physical and mechanical properties. In our previous study [26], G-ænial Universal Flo, in spite of being flowable, was a high performer in flexural strength when compared to other resin composites. To further explore and validate these results, we selected G-ænial Universal Flo in this study.

Filler content is an important factor affecting the physical and mechanical properties of resin composites. However, specifications provided by manufacturers regarding filler load have been shown to be inaccurate in certain instances [7]. Evaluating the filler content by ash method may provide more reliable measurement for research purposes. The results of our present study in regard to the filler weight measurements revealed lower filler weight percentage for all tested materials compared to the specifications released by the manufacturer (Table 1) which is in agreement with the findings of Kim et al. [7].

### 4.1. Fracture Toughness

Fracture toughness (KIc) is a basic property of materials, which indicates the resistance to crack propagation. K_Ic_ describes the critical intensity level at which a microdefect will lead to a catastrophic failure. A low fracture toughness is an indication of a high possibility of restoration failure under load [11, 27, 28]. Comparison of fracture toughness of the materials in wet and dry conditions revealed no difference between the conditions in most of the study groups. However, in AB, significantly lower toughness was observed in the wet condition compared to dry condition after 7 and 60 days. A similar reduction in fracture toughness of resin composites following aging in water has been previously reported by several studies [29–31]. Bagheri et al. [32] also reported a decrease in fracture toughness of resin composites stored in distilled water with the highest decrease after 8 weeks. In conclusion, it is safe to assume that the effect of storage condition on the fracture toughness was material dependent in the present study and the wet condition had no effect on the fracture toughness of TE, GUF, and GCK.

As the findings of the present study revealed, in all storage times and in both conditions, the fracture toughness of GUF was higher than other materials except for 24 h dry condition. The mean filler size has previously been reported to affect the fracture toughness of resin composites and those with smaller filler particles have been associated with higher toughness [33]. It has been shown that the reduced filler size could alter the organic matrix between the particles and decrease the interparticle distances, leading to improvements in mechanical properties. G-ænial Universal Flo contains small filler particles (SiO_2_ (16 nm), Sr glass (200 nm) with approximately 63 wt. %. In fact, this flowable resin composite is produced by incorporating the same small particle size of traditional hybrid composites, while at the same time reducing the filler content and increasing the resin to reduce the viscosity of the mixture [34]. Thus, the greater fracture toughness value of GUF observed in the present study may be attributed to its smaller particle size. Moreover, in the present study, resin composites with prepolymerized particles (AB, TE, and GCK) exhibited lower fracture toughness values compared to GUF. In line with our findings, Kim et al. [7] also reported that composites which contained prepolymerized particles had significantly low fracture toughness.

In addition, the results showed a strong inverse correlation between fracture toughness and filler weight. Thus, according to our findings, the lower filler weight of GUF can be another possible factor leading to its higher fracture toughness. As filler weight increased, K_Ic_ decreased, likely due to an exacerbated concentration of filler agglomerates in the material consequent to a rise in viscosity [33]. However, this finding disagrees with other studies [7, 35] on the effect of filler vol % on fracture toughness, which showed that the fracture toughness of composites increases as filler volume fraction is increased. One of the limitations of our study was that we were unable to measure the filler volume; instead we recorded the filler weight. Hence, further studies are required to investigate the influence of filler vol % on the physical and mechanical properties of resin composites in various storage conditions. In the clinical view, one of the criteria that recently suggested to be considered while measuring the fracture toughness of a tooth is size, shape, and microstructure of the tooth [36]. The authors concluded that reporting only the maximum FL may be misleading because the root surface area and volume made a significant difference to the outcome [36]. This difference can be explained by the complicity of fracture mechanics.

### 4.2. Vickers Microhardness

Vickers microhardness is a surface property that is defined as the resistance of the material surface to indentation [37]. Measuring the surface hardness can give an indication of the degree of conversion and consequently the clinical performance of resin composite material after aging in food simulating solvents [38, 39].

In the present study, for all time intervals in both conditions, there was a significant difference between the VHN of materials, with the highest value observed for GCK and AB and the lowest for GUF. Higher VHN has been reported to be correlated with lower porosity in the materials' structures [40]. Furthermore, the modified strontium glass, which is present in GCK, reinforces the filler's strength, offering improved surface hardness [20]. A previous study by Chinelatti et al. [41] confirms the result obtained in the present work indicating that flowable resin composites demonstrate lower microhardness compared to their conventional counterparts. In the present study, GUF had significantly lower filler weight (62.82%) compared to other resin composites. Thus, the lower VHN value of GUF can be explained by its lower filler weight. This finding is substantiated by those previous studies which reported a direct relationship between filler weight and surface hardness of resin-based materials [42, 43].

The data indicates that while the storage condition had a significant effect on the VHN of resin composites, the VHN was not subjected to aging. In fact, the VHN of the materials did not alter significantly after 60 days of storage compared to the first 7 days. The highest VHN was observed for GCK and AB, with GCK having higher values in wet and AB having higher values in dry condition. When compared to the dry condition, the lower VHN in the wet condition for the bulk-fill composite could be due to uptake of the aging liquid in porous intermolecular spaces within the resin composite, which leads to the physical destruction of the material [39]. Similar to our findings, Drummond et al. [44] showed that while the storage media has a significant influence on the resin composites, the storage time does not pose such effects. However, in a study by Hahnel et al., the authors proposed that both the composite materials and the storage times have a significant effect on the surface hardness [45].

### 4.3. Water Sorption/Solubility

The sorption and solubility values of the resin composites investigated in the present study were well below the maximum recommended value of 50 mg/mm^3^, according to the ISO standards for restorative resins. In the first 7 days, a significant difference in the water sorption values was found between various materials, with GUF showing the highest values. However, no significant difference in water sorption was observed between the materials after 60 days of storage. The immersion time has been shown to have an important role in water sorption of resin-based materials indicating that 7 days' immersion in distilled water is not sufficient for evaluating the strength of resin based materials [46].

In addition, a negative correlation has been shown between the sorption values and the amount of filler loading (*r*^2^ = 0.304), in agreement with previous studies [16, 47, 48]. A reduction in the filler wt. % will result in an increase in the polymeric matrix with consequent increase of water sorption, as it is a property which is primarily related to the polymeric phase. In the same manner, the higher water sorption values for GUF in the first 7 days could be explained based on its lower filler content compared to other tested materials. Braden and Clarke [49] and Øysaed and Ruyter [50] also reported a higher water uptake in composite materials with lower filler content.

Significant differences were observed between the water solubility values of the materials in various storage times, with TE having the lowest amount of solubility followed by GCK, AB, and GUF. A possible explanation for the lower rate of water solubility by TE could be the presence of polymerization modulator chemical groups in the resin matrix. This act to reduce the effect of polymerization shrinkage stress, which may affect the resistance of the material to moisture. Solubility of a resin based materials in water is mostly related to the leaching of free residual monomers, additives, and filler components [51, 52]. The solubility of most of the materials tested in the first 7 days of storage showed negative values. The negative values do not indicate that no solubility occurred in these materials. In fact, they may suggest the materials' low solubility, which may be explained by incomplete dehydration.

Consistent with previous studies [53, 54], a strong correlation was found between water sorption/solubility in our study (*r*^2^ = 0.840). This represents a two-way diffusion process in resin composites. Moreover, the storage period is another factor which has been shown to greatly affect the degree of water sorption and solubility and consequently the mechanical properties of a resin composite [11]. In the current study, 60 days of storage in water led to a significant increase of water sorption and solubility values in all tested materials. Hydrophilic monomers such as Bis‐GMA with hydroxyl groups could explain why these materials still showed an increase in water sorption after 60 days of water storage. Moreover, the TEGDMA present in the tested composites has an affinity to water due to the molecule's water compatible ether‐linkage structure [55]. This finding confirms that of a previous study [16] that reported an increase in the sorption of resin composite with increased period of storage. In line with our research, another study [21] assessing the hygroscopic dimensional changes during sorption and desorption cycles in five resin composites, a flowable, a universal, two microhybrids, and a posterior restorative composite, reported a significant dehydration shrinkage to a negative value for a flowable resin composite. Another study also revealed that flowable resin composite (Vertise Flow; Kerr Co) exhibited the greatest water sorption and hygroscopic expansion after 150 d [56].

### 4.4. Colour Stability

To investigate colour change, the Standard Commission Internationale de L'Eclairage (CIE *L*^*∗*^*a*^*∗*^*b*^*∗*^) was used to measure the three-dimensional colour change of the specimens. The results of the current study revealed that almost all colour change values were below 3.3 except for ∆*E*_2_ of GUF (4.01 ± 0.44) and TE (4.13 ± 0.52). This finding indicates that the colour changes of all of the resin composites used in this study after 60 days were clinically undetectable [57]. As the results revealed, in almost all materials, wet stored specimens revealed a higher value for ∆*E* than the dry specimens, with the finding being significant in bulk-fill composites. The water sorption/solubility, surface reactivity, and setting reaction are considered as the influencing factors in dental material colour susceptibility [58]. Thus, the greater colour change in the wet groups can be related to the water sorption in this condition.

There was no statistical difference between materials in ∆*E*_1_ kept in dry conditions. However, significant differences were found between materials in terms of ∆*E*_2_ and ∆*E*_3_ in both dry and wet conditions (*P* < 0.05) with the greatest ∆*E*_3_ being reported in AB. The differences observed in colour susceptibility among different resin composites may be due to their different compositions [59], setting reaction, and degree of polymerization which affects both water sorption and colour change of aesthetic restorative materials [60, 61].

Generally, it is speculated that each material should be used for a specific clinical need based on its properties. The correlation of the present findings to clinical studies evaluating the stability of resin composite properties in oral conditions should be further investigated.

## 5. Conclusions

Within the limitations of this study, the following conclusions were drawn: the type of the resin composite material, as well as the storage media and aging had an influence on the physical and mechanical properties of the resin composites. Filler weight also showed significant effects on the fracture toughness of the resin composites. In general, GUF showed the highest fracture toughness, water sorption/solubility, and the lowest hardness compared to other resin composites. After 60 days, the colour changes of all resin composites used in this study were clinically invisible. It is speculated that each material should be used for a specific clinical need based on their properties.

## Figures and Tables

**Table 1:** Description of all the resin composites used in the study and filler weight (%) measured by the ash method.

Resin composite	Type	Manufacturer	Resin matrix	Filler content (wt. %), type, size	Measured filler wt. % (%)	Lot number
Aura (bulk-fill)	Nanohybrid	SDI, VIC, Australia	UDMA, Bis-EMA, Bis-GMA, TEGDMA	Amorphous SiO_2_, Ba-Al-Si glass, prepolymerized filler (74.2 wt. %)	72.71	160841

Tetric Evoceram (bulk-fill)	Nanohybrid	Ivoclar vivadent AG, Liechtenstein	Bis-GMA, Bis-EMA, UDMA	Ba-Al-Si glass, prepolymer filler (monomer, glass filler and ytterbium fluoride), spherical mixed oxide (79–81 wt. %)	72.74	V23650

G-ænial Universal Flo	Nanohybrid	GC corporation, Tokyo, Japan	UDMA, Bis-EMA, TEGDMA, pigment photo-initiator	SiO_2_ (16 nm), Sr glass (200 nm), (69 wt. %)	62.82	1606207

GC Kalore	Nanohybrid	GC corporation, Tokyo, Japan	DX-511 (DuPont monomer), UDMA, Bis-EMA	High-density radiopaque prepolymerized fillers, 400 nm modified Sr glass (80 wt. %)	71.25	1601201

Bis-GMA: bisphenol A glycidilmethacrylate; TEGDMA: triethyleneglycol dimethacrylate; Bis-EMA: ethoxylatedbis-phenol-A-dimethacrylate; UDMA: urethane dimethacrylate.

**Table 2:** Comparison of mean ± SD values of fracture toughness (*K*_Ic_) between all materials after wet or dry storage, analysed by independent *t*-test.

Storage time	1 day			7 days			60 days		
Condition	Dry	Wet	*P* value^*∗*^	Dry	Wet	*P* value^*∗*^	Dry	Wet	*P* value^*∗*^
Aura (bulk-fill)	16.80 ± 1.10^A^	17.74 ± 1.43A	0.235	17.76 ± 1.82^A^	15.16 ± 0.78^A^	0.009	16.82 ± 1.80^A^	13.55 ± 1.17^A^	0.001^*∗*^
Tetric Evoceram (bulk-fill)	15.87 ± 2.15^A^	17.10 ± 1.86A	0.959	14.39 ± 1.07^A^	14.88 ± 2.44^A^	0.686	18.31 ± 2.56^A^	15.32 ± 2.97^A^	0.107
G-ænial Universal Flo	22.97 ± 3.11^B^	19.25 ± 2.93A	0.074	23.4 ± 6.11^B^	22.09 ± 1.49^B^	0.630	27.94 ± 5.39^B^	26.58 ± 5.37^B^	0.671
GC Kalore	16.85 ± 0.86^A^	16.8 ± 2.01A	0.314	14.26 ± 1.77^A^	14.89 ± 3.14^A^	0.688	18.38 ± 2.64^A^	15.87 ± 3.49^A^	0.190
*P* value	<0.001	0.257		0.001	<0.001		<0.001	<0.001	

^*∗*^Significance level of independent *t*-test between wet and dry storage (*P* value< 0.05). Different uppercase letters show significant difference between materials in each condition (column).

**Table 3:** Comparison of mean ± SD values of Vickers microhardness between all materials after wet or dry storage, analysed by independent *t*-test.

Storage time	24 h			7 days			60 days		
Condition	Dry	Wet	*P* value^*∗*^	Dry	Wet	*P* value^*∗*^	Dry	Wet	*P* value^*∗*^
Aura (bulk-fill)	41.86 ± 4.13^C^	45.33 ± 1.90^A^	0.018	49.37 ± 2.15^A^	45.09 ± 2.60^A^	<0.001	51.41 ± 1.86^A^	48.01 ± 1.27^B^	<0.001
Tetric Evoceram (bulk-fill)	51.29 ± 2.76^A^	39.69 ± 2.93^B^	<0.001	44.63 ± 2.59^B^	41.49 ± 2.86^B^	0.010	45.60 ± 3.36^BC^	46.44 ± 3.37^B^	0.551
G-ænial Universal Flo	39.69 ± 2.37^C^	37.92 ± 3.76^B^	0.183	41.92 ± 1.69^C^	36.70 ± 1.79^C^	<0.001	42.28 ± 4.53^C^	42.32 ± 2.63^C^	0.977
GC Kalore	45.54 ± 2.50^B^	47.42 ± 1.36^A^	0.036	48.79 ± 2.87^A^	46.83 ± 1.53^A^	0.049	47.20 ± 3.27^B^	51.35 ± 3.17^A^	0.005
*P* value	<0.001	<0.001		<0.001	<0.001		<0.001	<0.001	

^*∗*^Significance level of independent *t*-test between wet and dry storage (*P* value <0.05). Different uppercase letters show significant difference between materials in each condition (column).

**Table 4:** Comparison of mean ± SD values of sorption and solubility between all materials after 3 storage times, analysed by ANOVA test.

	Sorption			Solubility		
	1 day	7 days	60 days	1 day	7 days	60 days
Aura (bulk-fill)	0.03 ± 0.02^A^	10.01 ± 2.29^A^	17.93 ± 0.91^A^	−8.66 ± 2.26^A^	−3.02 ± 1.22^A^	0.82 ± 0.71^A^
Tetric Evoceram (bulk-fill)	−12.83 ± 2.00^B^	12.16 ± 2.80^B^	26.66 ± 1.34^A^	−20.06 ± 3.04^A^	−8.80 ± 3.04^A^	−4.02 ± 2.52^A^
G-ænial Universal Flo	0.13 ± 0.09^A^	14.23 ± 1.40^C^	22.65 ± 9.83^A^	−6.73 ± 1.60^A^	1.24 ± 1.10^B^	5.58 ± 1.46^B^
GC Kalore	−6.04 ± 2.30^C^	3.80 ± 1.72^A^	19.62 ± 2 .87^A^	−9.86 ± 3.65^B^	−4.09 ± 1.37^C^	1.10 ± 0.67^C^
*P* value	<0.001	<0.001	0.259	<0.001	<0.001	<0.001

Different uppercase letters show significant difference between materials in each storage time (column).

**Table 5:** Mean (±SD) colour change values of different materials kept dry and wet in all time intervals.

Material	∆*E*_1_			∆*E*_2_			∆*E*_3_		
	Dry	Wet	*P* value	Dry	Wet	*P* value	Dry	Wet	*P* value
Aura (bulk-fill)	2.5 ± 0.7^a^	3.3 ± 0.27^ab^	0.041	0.9 ± 0.37^c^	1.74 ± 0.42^b^	0.007	2.29 ± 0.7^a^	2.83 ± 0.46^a^	0.174
GC Kalore	1.69 ± 0.58^a^	1.89 ± 1.04^b^	0.702	1.84 ± 0.77^bc^	2.68 ± 1.51^ab^	0.275	0.6 ± 0.29^c^	1.16 ± 0.91^b^	0.313
G-ænial Universal Flo	2.28 ± 0.07^a^	2.97 ± 1.46^b^	0.350	4.01 ± 0.44^a^	3.31 ± 1.2^ab^	0.218	1.97 ± 0.21^ab^	1.26 ± 0.32^b^	0.002
Tetric Evoceram (bulk-fill)	2.21 ± 1.01^a^	5.29 ± 1.5^a^	0.003	1.94 ± 0.72^b^	4.13 ± 0.52^a^	0.000	1.6 ± 0.18^b^	1.67 ± 0.87^ab^	0.864
*P* value	0.248	0.005	—	0.000	0.011	—	0.000	0.006	—

Different lowercase letters show significant difference between materials in each condition (column).

**Table 6:** Correlations between five measured parameters by the Pearson test.

		Sorption	Solubility	Colour change	VHN	Fracture toughness
Filler weight	Correlation coefficient	−0.052	0.462	0.477	0.304	−0.777^*∗∗*^
	*P* value	0.873	0.130	0.472	0.337	0.003
Fracture toughness	Correlation coefficient	0.210	−0.274	−0.036	−0.023	
	*P* value	0.512	0.389	0.836	0.944	
Microhardness	Correlation coefficient	0.243	0.223	0.126		
	*P* value	0.446	0.487	0.726		
Colour change	Correlation coefficient	0.430	0.244			
	*P* value	0.078	0.183			
Solubility	Correlation coefficient	0.840^*∗∗*^				
	*P* value	0.001				

## Data Availability

All data are available with the corresponding author and will be released upon request.
